# Rapid prediction of yellow tea free amino acids with hyperspectral images

**DOI:** 10.1371/journal.pone.0210084

**Published:** 2019-02-20

**Authors:** Baohua Yang, Yuan Gao, Hongmin Li, Shengbo Ye, Hongxia He, Shenru Xie

**Affiliations:** 1 School of Information and Computer, Anhui Agricultural University, Hefei, Anhui, China; 2 Key Laboratory of Agricultural IoT Technology Integration and Application, Ministry of Agriculture, Hefei, Anhui, China; 3 National Engineering and Technology Center for Information Agriculture (NETCIA), Nanjing Agricultural University, Nanjing, Jiangsu, China; 4 Department of Computer Science, Kansas State University, Manhattan, KS, United States of America; Agricultural University of Athens, GREECE

## Abstract

Free amino acids are an important indicator of the freshness of yellow tea. This study investigated a novel procedure for predicting the free amino acid (FAA) concentration of yellow tea. It was developed based on the combined spectral and textural features from hyperspectral images. For the purposes of exploration and comparison, hyperspectral images of yellow tea (150 samples) were captured and analyzed. The raw spectra were preprocessed with Savitzky-Golay (SG) smoothing. To reduce the dimension of spectral data, five feature wavelengths were extracted using the successive projections algorithm (SPA). Five textural features (angular second moment, entropy, contrast, correlation, and homogeneity) were extracted as textural variables from the characteristic grayscale images of the five characteristic wavelengths using the gray-level co-occurrence matrix (GLCM). The FAA content prediction model with different variables was established by a genetic algorithm-support vector regression (GA-SVR) algorithm. The results showed that better prediction results were obtained by combining the feature wavelengths and textural variables. Compared with other data, this prediction result was still very satisfactory in the GA-SVR model, indicating that data fusion was an effective way to enhance hyperspectral imaging ability for the determination of free amino acid values in yellow tea.

## 1. Introduction

Tea is one of the world's three most popular drinks.[1] As important chemical components of tea, amino acids not only determine the taste and quality of the tea[2–4] but also provide many health benefits as necessary human nutrients.[5–7] Many studies have focused on the analysis of amino acids in red tea, black tea or green tea.[8–10] There is very little research on yellow tea, a traditional Chinese tea that many people like to drink.[11] The amount of free amino acids (FAA) in yellow tea is an important index of the freshness, taste and aroma of yellow tea. Hence, in this study, we focus on building prediction models based on hyperspectral images to predict the amount FAA in yellow tea.

Hyperspectral images are three-dimensional blocks of data cubes with a series of images at different wavelengths, and they have two spatial dimensions and one spectral dimension. Hyperspectral imaging is nondestructive, combining the advantages of traditional imaging and spectroscopy techniques to obtain spatial and spectral information of detected objects simultaneously. It can simultaneously obtain all continuous spectral information for each pixel and continuous image information for each spectral band. [12] The spectral information can reflect the chemical composition and organizational structure of the sample, and image information can reflect the spatial distribution of samples, external attributes and geometric structure. Many researchers have attempted to visualize the chemical concentration of hyperspectral images using a nonlinear correction model, such as the back-propagation neutral network (BP-NN) algorithm,[13] the self-organizing map algorithm,[14] the random-frog algorithm,[15,16] radial basis function support vector regression (RBF-SVR) algorithms,[17] the least squares-support vector machine (LS-SVM) algorithm and the adaptive boosting (AdaBoost) algorithm.[18–20]

An objective and nondestructive technique would have many applications in the analysis of tea, such as different tea classifications and quality testing. [21–25] Xie et al. (2004) used this technique to measure the color components of tea with different drying periods. [26] Deng et al. (2015) used hyperspectral imaging to predict the moisture content of longjing tea. [27] Zhao et al. (2011) demonstrated that the chlorophyll content and distribution in tea leaf can be measured by hyperspectral imaging. [28] These findings have shown that hyperspectral imaging provides an objective and reliable technique for tea analysis. It can not only overcome the shortcomings of spectral information and image information in the fusion process but also take into account both the external and internal quality testing of tea at the same time.

In this study, we proposed a hyperspectral imaging technique-based method to predict FAA in yellow tea. A hyperspectral imaging system was built to acquire hyperspectral images of yellow tea samples. We first obtained spectral information from these images, i.e., the feature wavelengths were extracted with successive projections algorithms (SPA), and the texture was extracted from the images of five feature wavelengths. Second, prediction models based on genetic algorithm-support vector regression (GA-SVR) were constructed using different data fusions of spectral and textural features. Finally, we evaluated these models with two measurements: coefficient of determination (*R*^*2*^) and root mean square error (RMSE). It was found that the GA-SVR-based model combining spectral and textural information together achieved the best results among the models. The main contributions of this work are as follows. (1) We focused on the poorly studied problem about free amino acid analysis in yellow tea with a hyperspectral imaging system. (2) We built and evaluated models using different data fusions to predict the free amino acid amount in yellow tea. Specifically, SPA was used to extract five feature wavelengths of spectral information in hyperspectral images of yellow tea samples, and a gray-level co-occurrence matrix (GLCM) was used to generate the textural features from these five feature wavelengths images. (3) We achieved better prediction results using the GA-SVR model with data fusion, which provided a possible method for predicting the amount of FAA in yellow tea.

## 2. Materials and methods

**Fig 1:**
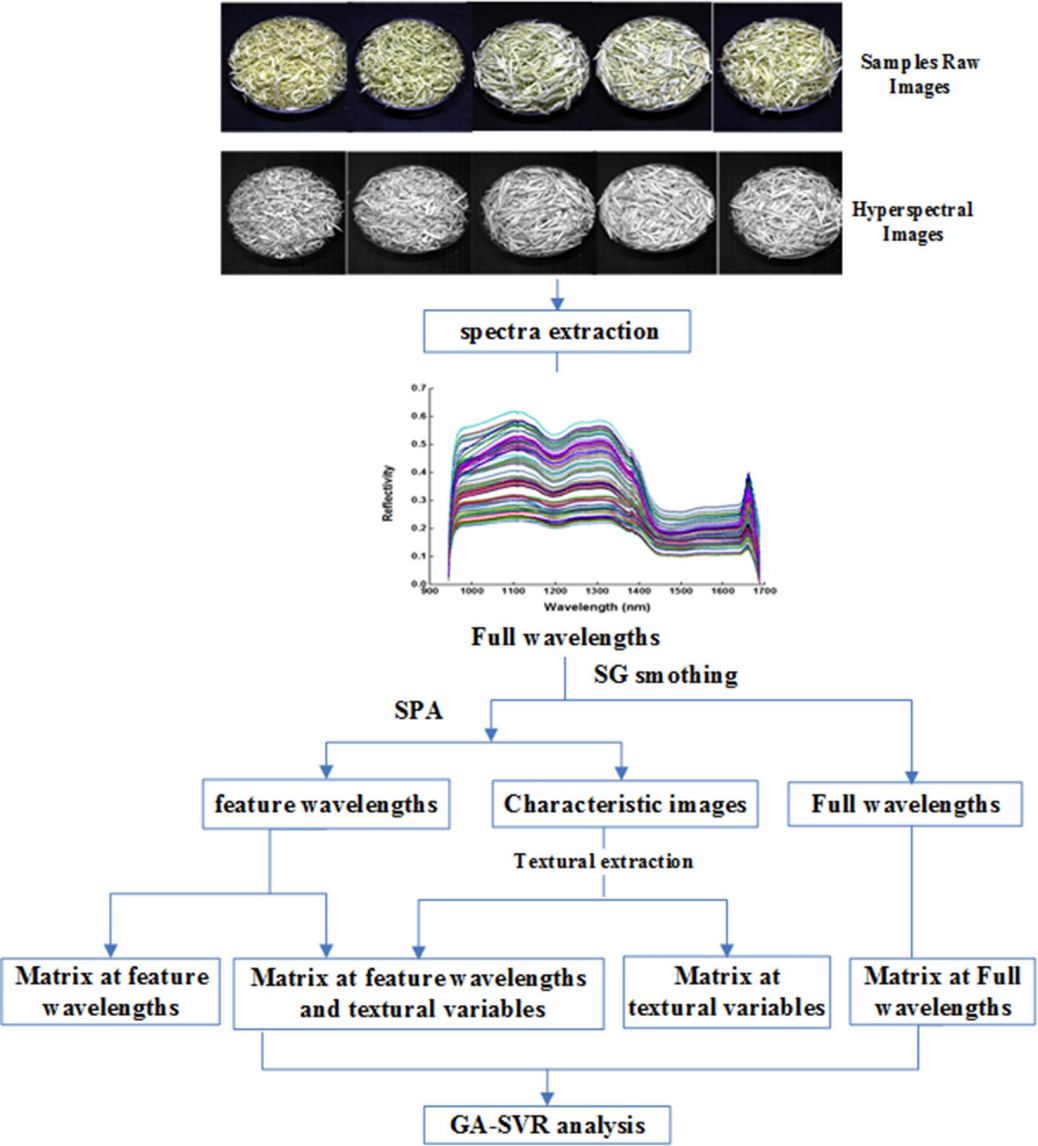
Flowchart of main data-processing procedures to predict FAA with hyperspectral images.

The main data-processing procedures for predicting FAA value in yellow tea by our hyperspectral imaging system are presented in Fig 1. According to Chinese national standard GB/T8314-2013, the amount of FAA in yellow tea should be measured using the ninhydrin colorimetric method. The amount of FAA in tea is expressed as dry mass percentage (%), calculated according to following formula:

$$Amount=\frac{c/1000\times v_{1}/v_{2}}{m\times \omega }\times 100\%$$ (1)

where *c* is the amount of theanine and glutamic acid in milligrams (mg); *v*_*1*_ is the total amount of solution in milliliters (ml); *v*_*2*_ is the amount of test solution in milliliters (ml); *m* is the amount of solution in grams (g); ω is sample dry matter (%).

### 2.1 Yellow tea samples

Five typical yellow tea samples were purchased from the local market in Anhui, China, and were treated as experimental materials in this work, including Pingyang huangtang (PY), Mogan huangya (MG), Huoshan huangya (HS), Mengding huangya (MD), and Junshan yinzhen (JS). Their places of production were as follows: PY was from Pingyang of Zhejiang Province; MG was produced in Deqing, Zhejiang Province; HS was produced in Huoshan, Anhui Province; MD was produced in Mingshan, Sichuan Province; and JS was produced in Yueyang, Hunan Province. We had 30 samples of each of them, for a total of 150 samples. All teas were produced in 2017. All samples were dried in a forced-air oven at 50°C (Shanghai Yiheng Machinery Co., Ltd., Shanghai, China) for approximately 2 hours. To minimize the oxidation rate and aging of yellow tea under high-temperature or normal-temperature conditions, the yellow tea was packaged into a sealed plastic bag and randomly divided into 5 groups (30 samples per group), which were stored at 5 ± 1°C for 30 days. Among the 150 samples, 100 samples were randomly selected to construct a calibration model, and the remaining 50 samples were used to establish a prediction model.

### 2.2 Hyperspectral imaging system and image acquisition

**Fig 2:**
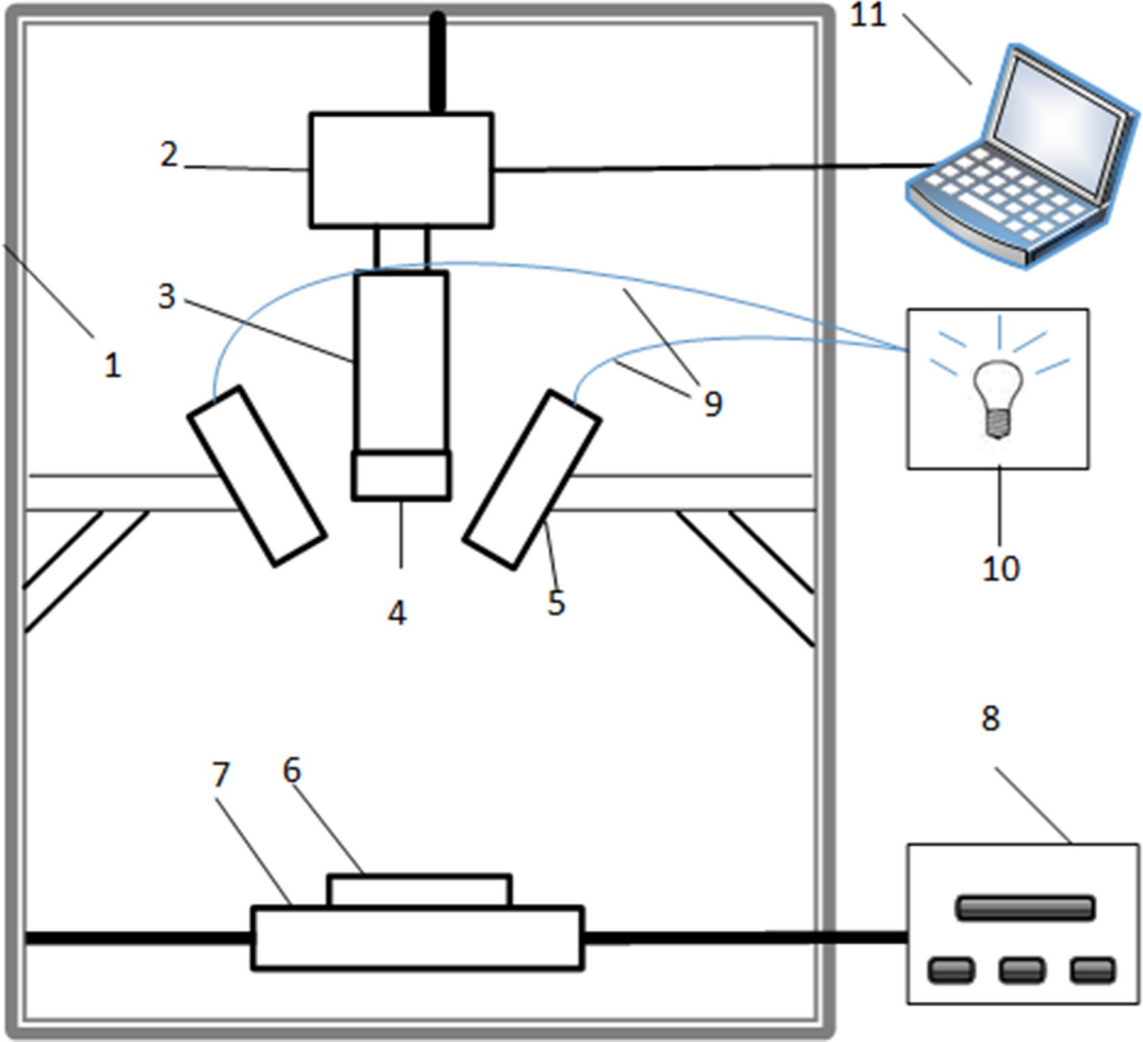
The hyperspectral imaging system. 1 dark room; 2 CCD camera; 3 imaging spectrograph; 4 lens; 5 light source; 6 Sample stage; 7 mobile platform; 8 mobile platform controller; 9 fiber; 10 light source controller; 11 computer.

Hyperspectral imaging is a combination of visible/near-infrared spectroscopy techniques and vision techniques, as shown in Fig 2. It is also known as imaging spectroscopy. [29] The hyperspectral imaging system contained a spectrograph (Imspector V17E, Spectral Imaging Ltd., Oulu, Finland), a charge-coupled device (CCD) camera (Hamamatsu, Japan), two 150-W tungsten halogen lamps for illumination (3900, Illumination Technologies Inc., New York, USA), a mobile platform, a black box, a computer for data collection, a conveyer belt (MTS120, Beijing Optical Instrument Factory, China), image acquisition and preprocessing software (Spectral Image Software, Isuzu Optics Corp., Taiwan, China).

Typically, when the beam is reflected from the sample and enters the objective lens, it is separated into its component wavelengths by the diffractive optical element contained in the diffractive optics. Then, a two-dimensional image (space size—wavelength size) is formed on the camera and stored on the computer. The sample is moved through the objective lens on the electric platform, and the process is repeated. The two-dimensional line images obtained at adjacent points on the object are stacked to form a three-dimensional hypercube that can be stored on the PC for further analysis. [30] To obtain the hyperspectral images of a yellow tea sample, 20 ± 0.5 g dry tea samples were collected and put evenly on a laboratory tray of size 9 cm×1 cm. The exposure time of the camera was set as 2 ms to ensure the clarity of the collected images. The speed of the conveyor was preset at 8 mm/s to avoid image-size and spatial resolution distortion. The vertical distance between the lens and the sample was 28 cm. Due to the presence of dark current noise and the nonuniform illumination, hyperspectral images of a sample collected under weak light waves contain a large amount of noise. Therefore, in this study, to eliminate the difference in illumination and detector sensitivity, the original raw hyperspectral image was calibrated to reflection mode and saved in the original format for further analysis. The raw hyperspectral image was calibrated with a black reference and a white reference before the data analysis. The whole black calibration image was obtained by completely closing the shutter of the camera. The white calibration image was obtained by opening the shutter and scanning a standard white correction board. Then, the raw images were converted into corrected images as follows:

$$R=\frac{I_{\mathrm{raw}}-I_{black}}{I_{\mathrm{white}}-I_{black}}$$ (2)

where *R* is the corrected image, *I*_raw_ is the original hyperspectral image, *I*_*black*_ is the black image and *I*_white_ is the white reference image.

### 2.3. Feature extraction

#### 2.3.1. Mean reflectance spectra extraction and preprocessing

**Fig 3:**
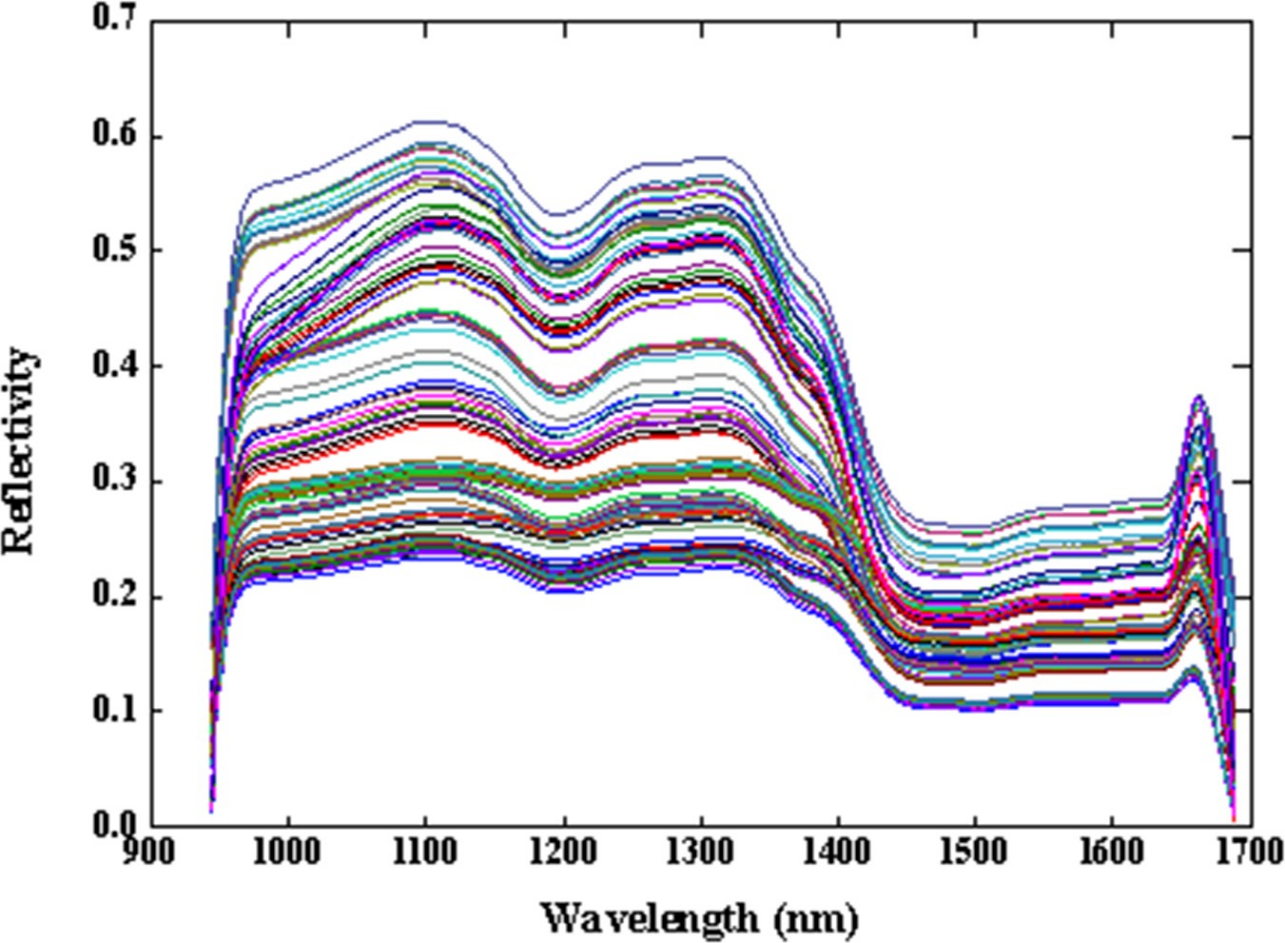
Full spectrum of yellow tea preprocessed by SG smoothing filter.

After acquiring and calibrating the hyperspectral image, the region of interest (ROI) was separated from the yellow tea sample, and the average spectral data within the ROI were manually extracted using the software ENVI version 4.8 (ITT Visual Information Solutions, Boulder, CO, USA). The extracted spectrum of the 40x40 region selected from each image was the average spectrum of the sample. A total of 150 samples of hyperspectral images were extracted. The raw average spectra obtained were in the range of 908–1735 nm, which altogether contained 508 wavelengths. However, in the process of hyperspectral imaging, the collected raw data often contained various noise due to interference from the acquisition environment, sensor noise, and other uncertainties. Only 944–1688 nm was considered to be valid, so 457 wavelengths were selected for further analysis, and the obtained spectra were combined into the spectral matrix (150 samples×457 wavelengths). Since spectral acquisition is affected by factors such as temperature, the original spectral data may have contained adverse effects from high-frequency random noise, sample inhomogeneity, baseline drift, and light scattering. Therefore, to reduce the baseline offset and eliminate random noise, the spectra were preprocessed by a Savitzky-Golay (SG) smoothing filter before selecting the feature wavelengths (Fig 3). However, in SG smoothing, if the smoothing window is too small and the denoising effect is poor, it will still affect the quality of the analysis model. If the window is too large and smooth, it will lose too much spectral information. For these reasons, the frame size and polynomial order must be specified. The frame size must be odd and was set to 21, and the polynomial order must be less than the frame length and was set to 3 in this study.

#### 2.3.2 Features wavelength selection

A hyperspectral image contains both one-dimensional spectral information and two-dimensional image information. There are usually redundant information and collinear problems in the direct analysis of the raw hyperspectral images. Therefore, feature wavelength selection was first performed during image processing. Feature wavelength selection not only helps to improve the efficiency of the processing by reducing calculation time but also helps to produce more simple and robust models. [31,32] The successive projections algorithm (SPA) is a forward-loop algorithm that uses vector projection analysis to select the variable group with the least redundant information through multiple iterations. As the collinearity between variables is minimized, the speed and efficiency of the model are improved. [27, 32]

The successive projections algorithm (SPA) is a variable selection technique designed to minimize collinearity problems in multiple linear regression (MLR). The SPA was initially proposed by Araújo et al. for multivariate calibration analysis. The main purpose of the algorithm is to achieve the minimum redundancy of the selected wavelength. The main steps of the SPA can be summarized as follows, assuming that the first wavelength *k*(0) and the number *N* are given [33]:

Step 1: before the first iteration (*n* = 1), let *X*_*j*_ = *j*_*th*_ column of *X*_*cal*_; *j* = 1,2⋯,*J*;
Step 2: let *S* be the set of wavelengths that have not been selected yet. That is, *S* = {*j* such that 1≤*j*≤*J* and *j*∉{*k*(0),⋯*k*(*n*−1)}};
Step 3: calculate the projection of *x*_*j*_ on the subspace orthogonal to *x*_*k*(*n*−1)_ as:

$$px_{j}=x_{j}-\left(x_{j}^{T}x_{k\left(n-1\right)}\right)x_{k\left(n-1\right)}{\left(x_{k\left(n-1\right)}^{T}x_{k\left(n-1\right)}\right)}^{-1}$$ (3)

for all *j*∈*S*, where *P* is the projection operator;

Step 4:
  $$K\left(n\right)=\arg\left(\max\|Px_{j}\|,j\in S\right);x_{j}=Px_{j},j\in S;$$ (4)

Step 5: let *n* = *n*+1. If *n*<*N* go back to Step 1;

End: the resulting wavelengths are {*k*(*n*);*n* = 0⋯*N*−1}.

#### 2.3.3 Textural feature extraction

The gray-level co-occurrence matrix (GLCM) is an important method for analyzing the textural features of images based on the second-order combined conditional probability density function of the estimated image. It extracts textural features from the co-occurrence matrix with statistical methods. Many of the details of the GLCM protocol have been described. [34] In this paper, the five feature parameters with the strongest texture description function were quantified[35]:

GLCM correlation was quantified as:

$$S_{cor}=\frac{\sum_{i=0}^{L-1}\sum_{j=0}^{L-1}i,jp(i,j|d,\theta )-\mu_{1}\mu_{2}}{\sigma_{1}^{2}\sigma_{2}^{2}}$$ (5)

In the formula *μ*_1_, *μ*_2_, $\sigma_{1}^{2}$, $\sigma_{2}^{2}$, they are defined as:

$$\mu_{1}=\sum_{i=0}^{L-1}i\sum_{j=0}^{L-1}p(i,j|d,\theta ),\;\mu_{2}=\sum_{i=0}^{L-1}j\sum_{j=0}^{L-1}p(i,j|d,\theta )$$

$$\sigma_{1}^{2}=\sum_{i=0}^{L-1}{\left(i-\mu_{{}_{1}}\right)}^{2}\sum_{j=0}^{L-1}p(i,j|d,\theta ),\;\sigma_{1}^{2}=\sum_{j=0}^{L-1}{\left(j-\mu_{2}\right)}^{2}\sum_{i=0}^{L-1}p(i,j|d,\theta )$$

GLCM angular second moment was determined as:

$$S_{E}=\sum_{i=0}^{L-1}\sum_{j=0}^{L-1}{\left\{p(i,j|d,\theta )\right\}}^{2}$$ (6)

GLCM homogeneity was calculated according to:

$$S_{H}=\sum_{i=0}^{L-1}\sum_{j=0}^{L-1}\frac{{\left\{p(i,j|d,\theta )\right\}}^{{}^{2}}}{1+{\left(i-j\right)}^{2}}$$ (7)

GLCM contrast was calculated according to:

$$S_{con}=\sum_{i=0}^{L-1}\sum_{j=0}^{L-1}{\left(i-j\right)}^{2}p(i,j|d,\theta )$$ (8)

Finally, GLCM entropy was determined as:

$$S_{P}=-\sum_{i=0}^{L-1}\sum_{j=0}^{L-1}p(i,j|d,\theta )\log\;p(i,j|d,\theta )$$ (9)

where i and j are the grayscale values in the co-occurrence matrix, and μ is the mean and σ the standard deviation, which are determined from pixel pair *p*(*i*,*j*|*d*,*θ*). Here, the correlation (COR) is a measure of the similarity of spatial gray-level dependence matrix elements in a row or column, reflecting the local gray correlation in the image. When the matrix element values are nearly equal, the correlation value is large; conversely, if the matrix pixel values differ greatly, the correlation value is small. Angular second moment (ASM) is used to measure the degree of gray-scale stability of the image texture, which reflects the uniformity of the image gray distribution and texture thickness. If all values of the co-occurrence matrix are equal, the ASM value is small; conversely, if some of the values are large and the other values are small, the ASM value is large. Homogeneity (H) is the measure of the local change in image texture and describes the regularity of the texture. Contrast (CON) is used to measure the distribution of matrix values and local variations in the image, reflecting the sharpness of the image and the texture of the grooves. Entropy (ENT) is used to measure the amount of information that an image has, which reflects the degree of complexity or complexity of the texture in the image. In previous studies, feature images in hyperspectral data were selected by principal component analysis (PCA) conversion. Although some results were obtained, the results were general, probably because principal component analysis causes the original information to be lost. In this study, the images at the selected feature wavelengths were characteristic images. The textural information was extracted from characteristic images with GLCM.

### 2.4 Models and evaluation index

#### 2.4.1 Support vector regression

Support vector regression (SVR) is used to describe the regression of the support vector machine (SVM). SVR constructs a linear decision function in high-dimensional space to achieve linear regression, which can transform the original low-dimensional nonlinear problem into a high-dimensional space to give good solutions to complex multivariate equations and has been successfully applied to NIR spectroscopy prediction models. [36]

Assuming that the existing input samples are n-dimensional vectors, samples and corresponding output values (*x*_1_,*y*_1_),(*x*_2_,*y*_2_),…,(*x*_*k*_,*y*_*k*_), the regression problem is to find a mapping such that the corresponding values are found by mapping outside the sample. The basic principle of SVR is to transform the complex low-dimensional nonlinear regression problem into a linear regression of high-dimensional space through mapping. SVR aims to find the regression function, that is,

f(x)=wφ(x)+b (10)

where *w* is the weight vector and *b* is the threshold. The formula (11) can be transformed into the problem of minimizing the number of targets of the following formula, that is, *w* and *b*, by minimizing the following formula:

$$R(w)=min[\frac{1}{2}w^{T}w+C\sum_{i=1}^{n}\left(\xi_{i}+\xi_{i}^{*}\right)]$$ (11)

$$subject\;to\left\{ \begin{array}{l} y_{i}-f(x_{i})\leq \varepsilon +\xi_{i}\\ f(x_{i})-y_{i}\leq \varepsilon +\xi_{i}^{*}\\ \xi_{i},\xi_{i}^{*}\geq 0 \end{array}\right.$$ (12)

where *ξ* and ξ* are non-negative slack variables; *ε* is insensitive loss function parameters; and *C* represents punishment factors, whose role is to find a balance between empirical risk and model complexity.

Introducing the *Lagrangian* method can transform the above formula into its dual problem, namely:

$$J(\alpha_{i},\alpha_{i}^{*})=\max[\frac{1}{2}\sum_{i=1}^{n}\sum_{j=1}^{n}\left(\alpha_{i}-\alpha_{i}^{*}\right)(\alpha_{j}-\alpha_{j}^{*})]-K(x_{i},x_{j})+\sum_{i=1}^{n}\alpha_{i}^{*}(y_{i}-\varepsilon )-\sum_{i=1}^{n}\alpha_{i}(y_{i}-\varepsilon )$$ (13)

$$\mathrm{subject}\;to\left\{ \begin{array}{l} \sum_{i=1}^{n}(\alpha_{i}-\alpha_{i}^{*})=0\\ 0\leq \alpha_{i},\alpha_{i}^{*}\leq C \end{array}\right.$$ (14)

where *K*(*x*_*i*_,*x*_*j*_) is the kernel function of SVR. Different kernel functions have different kernel function parameters. The kernel function used in this paper is a Gaussian kernel, namely

$$K(x_{i},x_{j})=\exp[-\frac{\|x_{i}-{x\|}^{2}}{2\sigma^{2}}]$$ (15)

where *σ* is the kernel width and is the only adjustable parameter in the Gaussian radial basis kernel function. Formula (16) is solved to get the SVR regression function, i.e.,

$$f(x)=\sum_{i=1}^{n}\left(\alpha_{i}-\alpha_{i}^{*}\right)K(x_{i},x_{j})+b$$ (16)

where *K*(*x*_*i*_,*x*_*j*_) is the kernel function of SVR. Different kernel functions have different kernel function parameters. In this paper, the Gaussian radial basis kernel function was chosen as the kernel function.

#### 2.4.2 GA for parameter selection of SVR models

Genetic algorithms are global optimization search algorithms based on natural selection and inheritance developed by Darwin. [37] It is a method of searching for optimal solutions by simulating natural evolutionary processes, which includes parameter encoding, initial population setting, fitness function design, genetic operation design, and control parameter setting. [38] Some parameters in SVR will be optimized by GA. In this study, a GA-SVR model was constructed to predict FAA in yellow tea to obtain good predictive performance.

The SVR model has three free parameters (C, ε, σ), which are determined by the user. Although determining these parameters is often an iterative process, these parameters greatly affect the performance of the SVR model. In this study, a genetic algorithm (GA) was applied to select the optimal parameters for the SVR model. Let $g=\frac{1}{2}\sigma^{2}$. Then there are three parameters C, ε, g in the SVR model. The main steps were as follows:

- Initialization: An initial chromosome population was randomly established, which represented the values of the parameters C, ε and g in the SVR model. The range of C was defined as [0, 100], the range of ε was defined as [0.0001, 0.01], and the range of g was defined as [0, 1000]. The largest evolutionary algebra was 200, and the maximum number of populations was 20.
- Evaluation of fitness function: Calculate the fitness function of each chromosome. In this study, the root mean square error (RMSE) was used as a fitness function.
- Choice: Select excellent chromosomes for reproduction.
- Crossover and variation: Genetic manipulation of selected individuals based on cross-mutation probability.
- Stop conditions: If the termination condition was satisfied, the individual with the highest fitness was output, and the optimal solution was obtained by decoding, and if it was not satisfied, the execution was repeated from steps b to d until the condition was satisfied.

#### 2.4.3 Evaluation index

To assess the accuracy of the established model, leave-one-out cross-validation was used to verify the established model. One test sample was removed each time in the calibration set, and then a new model was created to predict the removed model based on the remaining calibration samples.[39] This process was repeated for each sample, and finally, the model was applied to predict the FAA value of the new sample to provide a more realistic assessment of the performance of the model. We evaluated the models with the determination coefficient (*R2*) and root mean square error (RMSE) on the calibration set and prediction set, which were indicators of the average error in the analysis and are expressed in the original measurement units.[40] These indexes were also used to judge the consistency between the measured and the predicted values. The performance of the model was established by determining the calibration factor (R2c), prediction (R2p) and their corresponding calibration root mean square error (RMSEC) and prediction (RMSEP). In detail, R2 represents the ratio of the variance in the predictor variable (Y), which can be explained by the variance of the independent variable (X), and the values of RMSEC and RMSEP measure the regression fit and prediction during calibration. In general, relatively high R2 values and low RMSE values indicate the model has better performance. A reliable model is expected to have higher R2c and R2p values, close to 1, while the values of RMSEC and RMSEP would be closer to zero. [41]

RMSEC and RMSEP are defined as follows:

$$RMSEC=\sqrt{\frac{1}{n_{c}}{\sum_{i=1}^{n_{c}}\left(M_{i}^{\prime }-N_{i}\right)}^{2}}$$ (17)

$$RMSEP=\sqrt{\frac{1}{n_{p}}{\sum_{i=1}^{n_{p}}\left(M_{i}^{\prime }-N_{i}\right)}^{2}}$$ (18)

The correlation coefficient (R) is defined as follows:

$$R=\sqrt{1-\frac{\sum_{i=1}^{n}{\left(N_{i}-M_{i}^{\prime }\right)}^{2}}{{\sum_{i=1}^{n}\left(N_{i}-\bar{M_{i}}\right)}^{2}}}$$ (19)

where *n* is the number of samples $M_{i}^{'}{}_{}^{}$ and *N*_*i*_ are the predicted and measured values of the *i*^*th*^ observation, and $\bar{M}$ is the mean value of the calibration or prediction sets. All models, validations and evaluations were performed with MATLAB R2010b (The MathWorks Inc., Natick, MA, USA) for Windows 10.

## 3. Results and discussion

### 3.1 Free amino acid content of yellow tea

The contents of FAA in 100 samples used for the calibration model were different because FAA content is positively correlated with freshness. During the production of yellow tea, amino acids in tea are degraded and transformed to produce aroma components and other taste components. Therefore, the differences in processing technology of the five varieties of yellow tea caused amino acids to react and change differently. The free amino acid contents of different samples of the same variety of yellow tea were more evenly distributed. FAA are the main source of umami in tea. During the fermentation of yellow tea, the protein is hydrolyzed to form FAA. With the further oxidation of the fermentation, some FAA form the corresponding polymers, and the relative content of FAA in the yellow tea decreases. Therefore, the FAA of yellow tea is an important factor that reflects the quality and flavor of yellow tea, and an estimation model of FAA will help to further identify the quality of yellow tea.

### 3.2 Selection of feature wavelengths and textural variables

**Fig 4:**
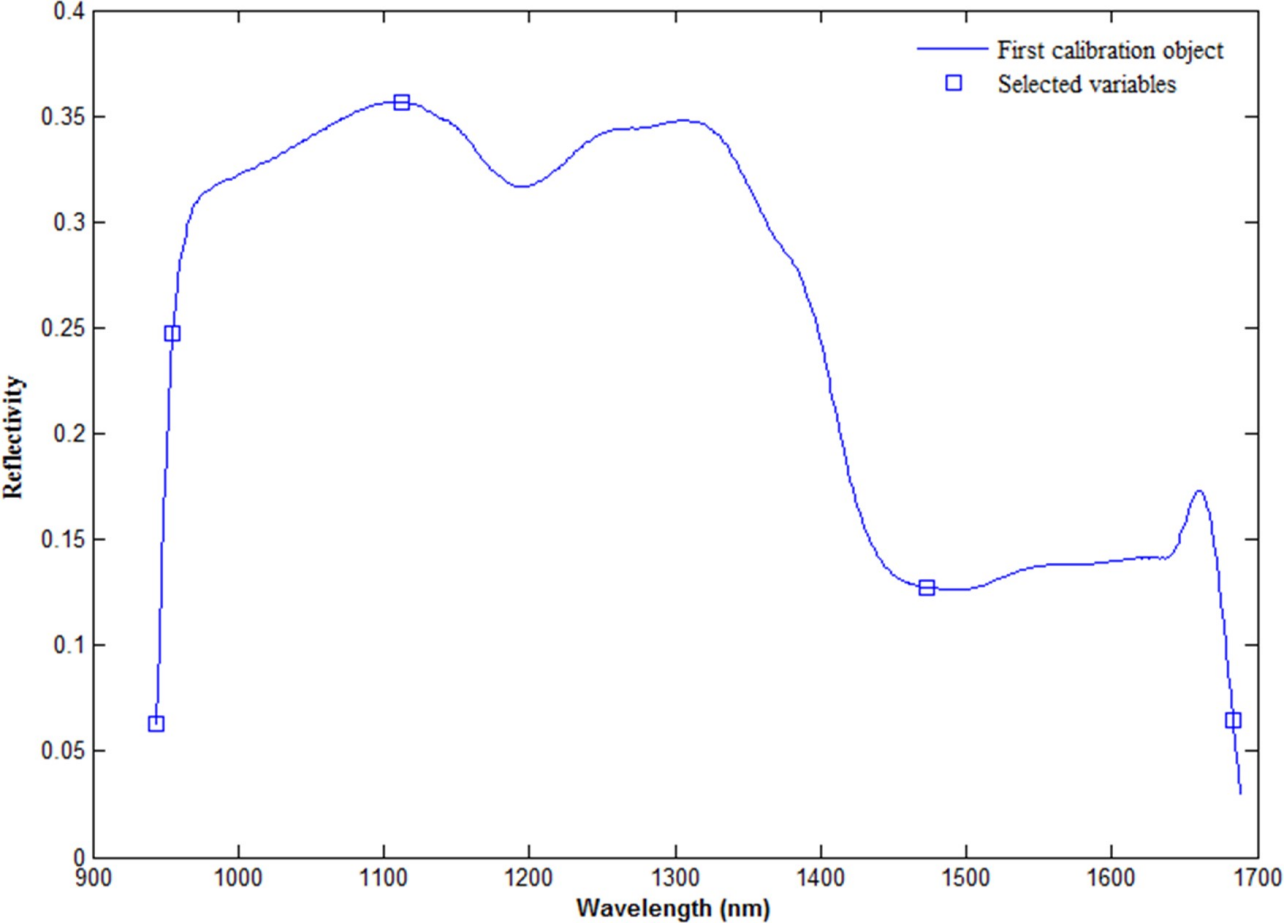
The selected feature wavelengths after SPA algorithm.

To decrease the dimensionality of the spectral data and reduce the computation time, SPA was employed to select the feature wavelengths from the whole spectral range. Then, SPA was used to extract the feature wavelengths from the spectral data of these 100 samples, and the ranges of SPA variables were set from one to ten. The root mean square error (RMSE) trends change when extracting different feature variables that are used to build the model based on partial least squares (PLS). [42] When the number of the selected feature variables increased from 2 to 5, the RMSE value dropped significantly, and the lowest value of it was 0.0166 when the selected variable number was 5. Therefore, five feature variables were selected as optimal spectral variables (944, 955, 1112, 1473, 1683 nm), as shown in Fig 4.

In addition, textural features were extracted from the feature grayscale images of the five feature wavelengths using GLCM. Yellow tea contains a large number of active ingredients related to amino acids. These active ingredients are mostly hydrogen-containing groups (C-H, O-H, N-H, etc.), which are absorbed at some specific wavelengths. Different varieties of yellow tea may have different active ingredient contents and proportions. These differences yield differences in the specific wavelength of light absorption intensity, that is, the performance of different spectral reflectance. Different varieties of yellow tea also have a certain relationship with the external quality of tea, which can describe the external quality of tea by five textural features, such as entropy, energy, correlation moment, moment of inertia and inverse gap. Thus, the gray value of the point on the selected sensitive wavelength image corresponds to the spectral value. According to formulas (6)–(10), the COR, ASM, H, CON and ENT at the angles of 0°, 45°, 90° and 135° in the corresponding image were calculated. As a result, 100 textural features variables were extracted.

### 3.3 Prediction of FAA values using spectral and textural variables

The GA-SVR model was used to predict the FAA value of yellow tea, wherein the independent variable FAA value was predicted by the full band variables, the feature wavelengths variables and the textural variables. Table 1 shows the main statistics used to evaluate the performance of the models built in the calibration and prediction procedures. It can be seen from Table 1 that the GA-SVR model based on the full-band showed poor results (R2p = 0.69, RMSEP = 18.81%), demonstrating the applicability of spectral features in predicting FAA values. The performance of the simplified GA-SVR model constructed by the five feature wavebands selected by the SPA was further improved, with R2p of 0.74 and RMSEP of 17.25%, indicating that the selected best bands were representative and could replace the full band for further prediction. The performance improvement may have been due to the SPA-selected band having minimal redundancy and containing most of the information related to the feature. [43] Compared with the model based on the feature wavelengths, although the performance of the model based on the textural variable was slightly improved, it also showed good prediction performance: R2p was 0.81, and RMSEP was 14.71%. Thus, spectral information can explain the chemical properties of yellow tea samples, which are closely related to FAA changes. Textural information usually represents the quality of different yellow teas, and textural features can also predict FAA values well.

**Table 1:** Performance of GA-SVR models based on different data for prediction of FAA.

Modeling data	Variables	Calibration set		Prediction set	
		R^2^_c_	RMSEC (%)	R^2^_p_	RMSEP (%)
Full Wavelengths	457	0.84	15.09	0.69	18.81
Feature Wavelengths	5	0.82	15.91	0.74	17.25
Texture variables	100	0.99	0.83	0.81	14.71
Data fusion	105	0.99	0.78	0.87	12.02

### 3.4 Enhanced prediction of FAA based on data fusion

Image fusion is an important part of image processing, whose aim is to fit the characteristics of spatial and spectral high resolution presented separately by the original images together in one image. [44] As discussed above, spectral and textural characteristics indicate their good ability to predict the free amino acid content of yellow tea. Therefore, spectral and textural features were integrated in the hyperspectral image to optimize predictive capability. Usually, the fusion process can be carried out at different levels and can be divided into signal level, pixel level, feature level, and decision level. Among them, signal-level image fusion is the optimal concentration or distribution detection problem of signals, which requires the highest registration time and space. Pixel-level fusion requires a large amount of data to be processed, and the time consumed when processing is relatively long, which is easily influenced by noise, and the data cannot be processed in real time. Decision-level fusion is the feature extraction of image data and the participation of some auxiliary information. This valuable information is combined to obtain comprehensive decision results to improve the recognition and interpretation capabilities. Feature-level fusion is the feature extraction of the original information from the sensor, followed by comprehensive analysis and processing of the feature information, which can keep more original information. Therefore, spectral and textural variables in hyperspectral images were merged at the feature level, and then the SVR-based prediction model of FAA in yellow tea was established based on data fusion of different features.

**Fig 5:**
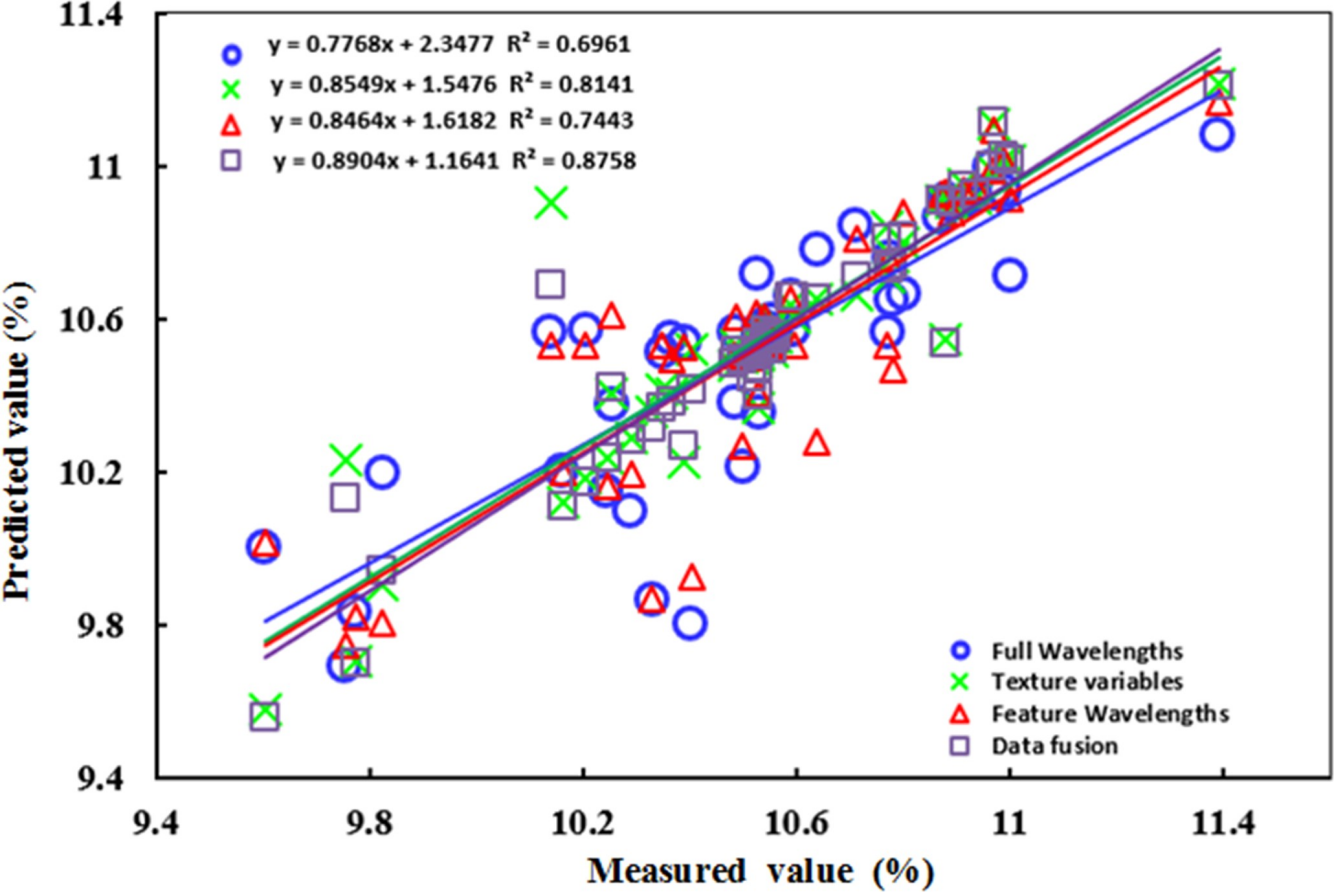
GA-SVR model between measured and predicted free amino acids based on fusion of different data.

Table 1 shows the main statistical indicators in the calibration set and the forecast set of two different kinds of fusion data. As seen from Table 1, the GA-SVR model built using the integrated spectrum and texture had good performance, possibly because the change in FAA in yellow tea was well reflected in the spectral and textural features of yellow tea. The spectral information can explain the chemical properties of the yellow tea sample that are closely related to changes in free amino acid-related compounds. As shown in Table 1, the model developed based on data fusion was more effective, and excellent results were obtained (R^2^_p_ = 0.87, RMSEP is 12.02%). Compared with the GA-SVR model using spectral data or textural data alone, the improvement was more than 10% in the R^2^_p_ value of the model, indicating that data fusion is an effective method to improve the hyperspectral imaging ability and determine the FAA value reflecting the yellow tea quality. Furthermore, to visualize the performance of the GA-SVR model, a linear fit between the measured FAA values obtained by the conventional method and the predicted values obtained by the corresponding GA-SVR models is shown in Fig 5. It can be clearly observed that the measured FAA value was best suited for FAA values predicted by the data fusion based on the GA-SVR model, probably because the data fusion simultaneously obtained the chemical and physical information of yellow tea, which completely explained the change in the FAA value of yellow tea.

## 4. Conclusion

In this study, the fusion of spectral and textural data improved the ability to quickly predict the FAA content of yellow tea using hyperspectral images. The quantitative GA-SVR models were established using the feature wavelengths (944, 955, 1112, 1473, 1683 nm) selected by SPA. The textural features were extracted from the characteristic images using GLCM at the selected wavelengths. The FAA content the prediction model with different combinations of variables was established by the genetic algorithm-support vector regression (GA-SVR) algorithm. After analysis and comparison, we found that the full-wavelength-based GA-SVR model and the feature wavelength-based GA-SVR model showed good performance in predicting free amino acids, with R^2^_p_ of 0.69 and RMSEP of 18.81%. The spectral and textural data were integrated by feature-level fusion. Our genetic algorithm-support vector regression model built based on data fusion yielded excellent results with a coefficient of 0.87. The performance of the model was improved when compared with the full wavelengths, the feature wavelengths or textural data alone. The results show that the method based on data fusion was effective for predicting the free amino acid content of yellow tea using hyperspectral imaging. The results of this work can facilitate the use of hyperspectral images to detect the free amino acid value of fresh tea online and improve the accuracy of the technique.

## Supporting information

S1 File Algorithms' source codes. (RAR)

S1 Table Spectral data of yellow tea. (XLSX)
